# Osteitis in the dens of axis caused by *Treponema pallidum*

**DOI:** 10.1186/1471-2334-13-347

**Published:** 2013-07-26

**Authors:** Thilde Fabricius, Charlotte Winther, Caroline Ewertsen, Michael Kemp, Susanne Dam Nielsen

**Affiliations:** 1 Department of Infectious Diseases, Copenhagen University Hospital, Rigshospitalet, Denmark; 2 Deparment of Infectious Diseases, Odense University Hospital, Rigshospitalet, Denmark; 3 Department of Pathology, Copenhagen University Hospital, Rigshospitalet, Denmark; 4 Department of Radiology, Copenhagen University Hospital, Rigshospitalet, Denmark; 5 Deparment of Microbiology, SSI, National Institute for Health Data and Disease Control, Copenhagen, Denmark

**Keywords:** *Syphilis*, *Osteitis*, *Treponema pallidum*, *HIV*, *PCR*

## Abstract

**Background:**

Syphilis has been referred to as “the great imitator” due to its ability to imitate other diseases. Untreated syphilis becomes a systemic infection that can involve almost every organ systems. *Treponema pallidum* has a high affinity for bone tissue, but osteitis has mainly been described in late stages of the disease. Vertebral involvement is rare, and this is to our knowledge the first case describing syphilitic spondylitis in early acquired syphilis.

**Case presentation:**

We here describe destructive osteitis in the vertebral column as the initial manifestation of early acquired syphilis in a 24-year-old caucasian homosexual male with HIV infection. The diagnosis was reached by universal bacterial PCR and DNA sequencing of the DNA product. It was confirmed by PCR specific for *Treponema pallidum*, immunohistochemistry and detection of increasing antibody titer.

**Conclusions:**

As syphilis has re-emerged in Western countries and remains a worldwide common disease it is important to have in mind as a causative agent of skeletal symptoms, especially among HIV-infected individuals or men who have sex with men (MSM).

## Background

Syphilis is a sexually transmitted disease caused by the spirochete *Treponema pallidum*. The presentation of the disease includes a variety of clinical symptoms as the spirochetes can be spread through the bloodstream to all organ systems. The disease is divided into early and late syphilis. Early syphilis includes a primary stage with a painless ulcer (chancre) and usually non-tender regional lymphadenopathy, and a secondary stage with multiorgan involvement due to bacteremia. Secondary syphilis occurs 4–8 weeks after inoculation where *T*. *pallidum* becomes a systemic infection. Bone involvement is a common finding in congenital and tertiary syphilis, but is seldom encountered in the early stage of syphilis. Syphilitic bone disease begins with seeding of the deeper vascular areas of periosteum by the spirochetes with resulting perivascular inflammatory infiltrates and subsequent formation of highly cellular granulation tissue. At this stage the pathologic process may regress or proceed to osteolytic or osteoblastic changes in the bones [1]. It is mainly the superficial bones that are involved (skull, sternum, tibia and clavicle).

Though treponemes have a pronounced affinity for bone tissue, osteitis is a rare manifestation of secondary syphilis [2-4]. The incidence of syphilis has had a dramatically increase during the last decade especially among men who have sex with men (MSM) and HIV-infected individuals [5-7]. With this resurgence it is important to have in mind that syphilis may present with unusual symptoms and clinical findings. To our knowledge this is the first case describing vertebral involvement in early syphilis.

## Case presentation

A 24-year-old HIV positive homosexual Danish male with no other chronic diseases was admitted to the hospital with four days of febrile episodes and thoracic pain with intermittent stabbing pain in both arms. He had also had bilateral leg pain worst at night time. He denied having had any kind of rash or genital ulcers. There had been no traumas in connection to the onset of pain.

Two weeks before admission, the patient contacted the Department of Infectious Diseases due to a sore throat. At this point physical examination was normal including a normal examination of the oral cavity. A throat culture was negative, and the patient did not have fever. The patient had been tested positive for HIV in 2002, and antiretroviral treatment with tenofovir/emtricitabine , atazanavir and ritonavir was initiated seven months prior to admission. Prior to treatment initiation the CD4 nadir was 330 cells/μl, and the HIV-1 RNA was 44549 copies/ml. Syphilis testing was performed at treatment initiation and 7 months before admission to the hospital, both serology tests were found negative.

**Figure 1:**
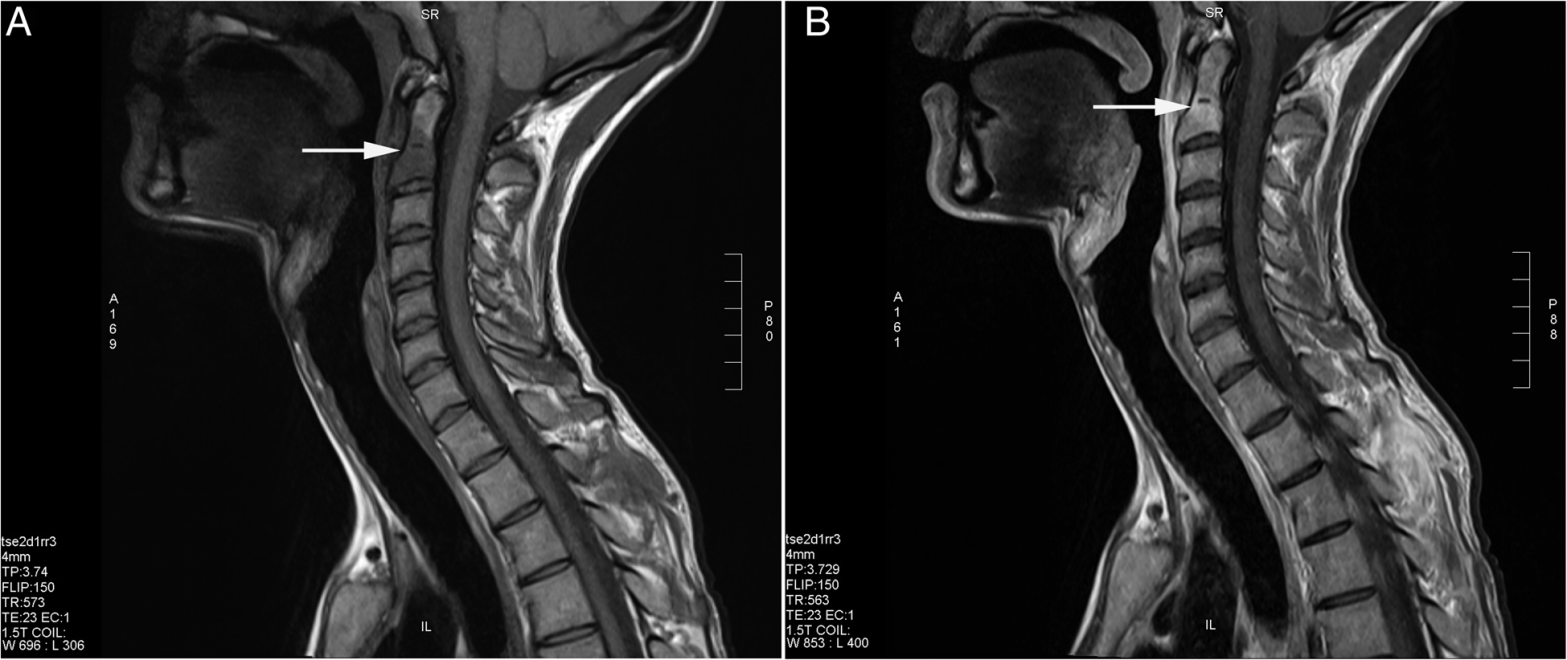
MRI images (T1 sequences) of the affected cervical vertebra (white arrows) before (A) and after (B) contrast enhancement.

On the admission day physical examination disclosed nothing abnormal except from severe tenderness of the processus spinosus of the second thoracic vertebrae (T2). The overlying skin was without any signs of infection and no rash or genital ulcers were found. Laboratory test demonstrated a normal white blood cell count 4.8 × 109/l and C-reactive protein level of 109 mg/l. The CD4 count was 360 cells/μl, and HIV-1 RNA was 158 copies/ml. A QuantiFERON-TB gold test was negative. Eight blood cultures were done, all negative for bacterial and fungal growth. Osteitis was suspected and a bone scintigraphy was performed which revealed bilateral tibial activity as well as abnormal activity in the right side of the cranium but no spine activity was observed. Magnetic resonance imaging (MRI) of the spine revealed destructive lesions in C2 (the dens of axis) and T2 consistent with osteitis (Figure 1).

**Figure 2:**
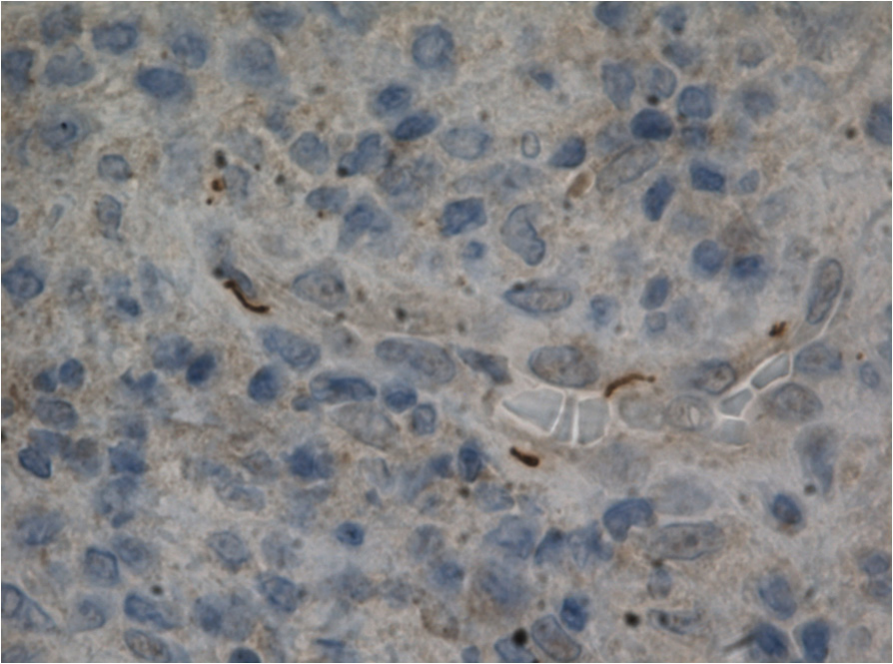
**Immunohistochemical analysis using polyclonal antibody against *T. pallidum *demonstrating several spirochetes.**

A surgical biopsy from T2 was performed, and culture from the biopsy was negative for bacterial growth. Acid-fast and Gram stain did not reveal any microorganisms. The biopsy was then analyzed for bacterial DNA. PCR for part of the 16S rRNA gene resulted in a 528 basepair fragment which by DNA sequencing and subsequent comparison to sequences deposited in the NCBI database showed sequence homology with *Treponema pallidum* (505 out of 507 identical bases). The bacterial identification was confirmed by PCR specific for the organism [8]. The pathological examination revealed an inflammatory infiltrate dominated by lymphocytes and plasmacells. Immunohistochemical analysis using polyclonal antibody against *T*. *pallidum* demonstrated several spirochetes (Figure 2). Furthermore, a reactive quantitative rapid plasma reagin (RPR) syphilis serology was found to be positive with a 1:128 titer. As soon as the biopsy was obtained treatment was initiated with cefuroxime 1.5 g × 3 daily. Following the diagnosis of syphilitic osteitis, treatment was changed to Ceftriaxone 2 g × 1 daily for five weeks according to the local guidelines. The thoracic pain disappeared within the first week of treatment. A follow-up MRI of the spine performed after two weeks of treatment revealed unaltered lesions.

## Conclusion

In the case presented *T*. *pallidum* was identified as the unexpected cause of a vertebral osteitis. Clinically significant osteitis and osteomyelitis are rare complications of secondary syphilis unlike bone involvement in congenital and tertiary syphilis.

The syphilitic bone lesions usually have origin in the periostitis but can spread to the subjacent bone. The most frequently affected bones are tibia and the skull.

In our case, an HIV positive male with early stage of syphilis, as he had a negative syphilis serology 7 months prior to admission had osteitis. There was no recognized chancre unless the throat pain represented an undiagnosed chancre. The nocturnal shin pain our patient described is a typical symptom of syphilitic periostitis and often accompanied by swelling and erythema. Thus, the findings on the scintigraphy with tibial and cranial activity are compatible with periostitis.

As the osseous lesions can be hard to encounter the prevalence of bone involvement in syphilis is unknown. In one study two patients out of 851 (0.2%) had periostitis [4]. In another x-ray study survey of the skulls of 80 patients with secondary syphilis, 7 patients (9%) had cranial lesions [9]. The by far largest study performed by Reynolds and Wasserman in 1942 found that only 15 patients out of 10.000 (0.15%) with syphilis had destructive bony changes [2].

During the last decades a number of cases of bone involvement in early syphilis have been reported [10-14]. In these cases the diagnosis has primarily been reached by radiological findings (bone scintigraphy, MRI, x-ray). Only one case of syphilitic osteitis where the diagnosis is obtained by PCR technique has been formerly reported [10]. In very few cases spirochetes have been isolated from bone biopsy [3]. Recently, increasing incidence of syphilis, especially among MSM, has been reported across Europe [5,6,15]. In Denmark, the number of reported cases has tripled from 2008 to 2009 [5] and the increase continues within 2010 [6]. In California there was a >700% increase in primary and secondary syphilis cases reported between 1999 and 2005, and 80% of these cases involved MSM. Furthermore 60% of MSM with syphilis were co-infected with HIV [7]. The increasing incidence of STD may indicate a decrease in the safe sex practice that may be due to the decreased awareness of HIV transmission after well established antiretroviral treatment. With this in mind syphilitic osteitis should be considered for at-risk patients with bone symptoms or with lytic bone lesions.

In conclusion we describe an atypical presentation of syphilis in a young HIV infected male with osteitis in vertebrae including the dens of axis caused by *T pallidum*. MRI changes lead to bone biopsy and subsequently the diagnosis of syphilis obtained by specific PCR technique. The rare location of a syphilitic osteitis and the diagnostic approach make this case unique.

## Consent

Written informed consent was obtained from the patient for publication of this case report and accompanying images.

## Abbreviations

HIV: Human immunodeficiency virus; PCR: Polymerase chain reaction; MSM: Men who have sex with men; HAART: Highly Active retrovirale treatment; STD: Sexual transmitted diseases; cART: Combination antiretroviral treatment; MRI: Magnetic resonance imaging.

## Competing interests

The authors declare that they have no competing interests.

## Authors’ contributions

TF and SDN were clinical responsible. CE responsible for CT–scan and description. CW Responsible forpathological examinations. MK responsible for 16S PCR and syphilis specific PCR. All authors have read the manuscript and accepted its final version.

## Pre-publication history

The pre-publication history for this paper can be accessed here:

http://www.biomedcentral.com/1471-2334/13/347/prepub
